# Ocular medicines in children: the regulatory situation related to clinical research

**DOI:** 10.1186/1471-2431-12-8

**Published:** 2012-01-20

**Authors:** Filomena Fortinguerra, Antonio Clavenna, Maurizio Bonati

**Affiliations:** 1 Laboratory for Mother and Child Health, Department of Public Health, Mario Negri Institute for Pharmacological Research, Milan, Italy

**Keywords:** review, ocular medicines, eye diseases, drug therapy, paediatrics

## Abstract

**Background:**

Many ocular medications are prescribed for paediatric patients, but the evidence for their rational use is very scant. This study was planned to compare the availability and the licensing status of ocular medications marketed in Italy, the United Kingdom (UK), and the United States of America (USA) related to the amount of published and un-published RCTs testing these drugs in the paediatric population.

**Methods:**

A quantitative analysis was performed to evaluate the number of ocular medications with a paediatric license in Italy, the UK, and the USA. A literature search was also performed in MEDLINE, EMBASE, and The Cochrane Central Register of Controlled Trials for randomized controlled trials (RCTs) on ophthalmic pharmacological therapy in children aged < 18 years, published up to December 2010. A search in the international clinical trial registries, the list of paediatric investigation plans (PIPs) approved by European Medicines Agency (EMA), and the table of medicines with new paediatric information approved by Food and Drug Administration (FDA) was also performed.

**Results:**

In all, of 197 drugs identified, 68 (35%) single drugs are licensed for paediatric use at least in one considered country, while 23 (12%) were marketed in all three countries. More specifically, in Italy 43 single drugs (48% of those marketed) had a paediatric license, while 39 (64%) did in the UK and 22 (54%) did in the USA. Only 13 drugs were marketed with a paediatric license in all countries.

The percentage of drugs licensed for paediatric use and for which at least one RCT had been performed ranged between 51% in Italy and 55% in the USA. No published RCTs were found for 11 (48%) drugs licensed for paediatric use in all three countries. In all, 74 (35%) of the retrieved RCTs involved mydriatic/cycloplegic medications.

A total of 62 RCTs (56% completed) on 46 drugs were found in the international clinical trial registries. Cyclosporin and bevacizumab were being studied in many ongoing trials. Twenty-six drugs had new paediatric information approved by FDA based on new paediatric clinical trials, while only 4 PIPs were approved by EMA.

**Conclusions:**

There is a pressing need for further research and clinical development in the pediatric ophthalmic area, where effective up-to-date treatments, and additional research and education on use in children, remain priorities.

## Background

Many drugs on the market labelled for adult use contain no information on paediatric use because their safety and efficacy have not been well studied in paediatric patients [1]. Many widely used drugs therefore include disclaimers stating that the paediatric use is "not recommended". Despite the prevalence of eye disease in early childhood (in the United Kingdom, by 3 years of age 5.7% of children had had ≥ 1 eye condition, 0.24% of which associated with visual impairment) [2] more than in other paediatric areas, evidence for the rational use of ocular medicines in these patients is very scant.

Many ocular medications are used in children to treat common bacterial and viral infections, inflammation and allergy, uveitis and glaucoma, as well as other conditions including myopia, amblyopia, and strabismus [3], even if data regarding their safety and effectiveness in the paediatric population are sparse. In 2000, a review of the 98 most commonly used or prescribed topical ophthalmic drugs found that only 51% provided information on paediatric use [4]. Without adequate paediatric labelling information, practitioners treating eye disease in children may be forced to prescribe ocular medications in an "off-label" manner, placing their paediatric patients at risk for serious adverse reactions [5,6].

Children are not small adults. Statements regarding paediatric drug use must be age-specific to indicate for which group a drug has been studied: newborns, infants, pre-school children, school-age children, and adolescents. These groups differ not only in size and body weight but in physiology and metabolism as well [7]. Children, in particular infants and neonates who have thin eye membranes, may be particularly vulnerable to systemic effects of topical ophthalmic drugs as the doses used are often not weight-adjusted and are similar to doses used in adults. Systemic absorption may have a greater impact in children than in adults due to their lower body mass, altered metabolic capacity, and an immature blood brain barrier, leading to potentially higher plasma levels for a longer period of time and to a much greater risk of serious systemic side effects [8].

In addition to these differences, other characteristics unique to the paediatric population include the lack of commercially available dosage forms and concentrations appropriate for paediatric patients and the lack of published research on the pharmacokinetics and clinical use of new drugs [9]. The result is the high frequency of serious medication errors.

A study was planned to compare the availability and the licensing status of ocular medications marketed in Italy, the United Kingdom (UK), and the United States of America (USA) related to the amount of published and un-published RCTs testing these drugs in the paediatric population.

## Methods

Ocular medications were identified and classified according to the International Anatomic-Therapeutical-Chemical classification system (ATC) [10] as S01: antibiotics, antivirals, anti-allergy drugs, non-steroidal anti-inflammatory drugs (NSAIDs), steroids, diagnostic agents, lubricants, glaucoma medications, local anaesthetics, and vascular endothelial growth factor inhibitors (anti-VEGF drugs) and combinations. A quantitative analysis was performed to record the number of ophthalmic drugs available on the market and those approved for paediatric use in Italy, the UK, and USA. Data on the licensing status of individual drugs were obtained by consulting national formularies: Italy's Repertorio Farmaceutico Italiano (Refi) [11], the UK's British National Formulary (BNF) [12], and the USA's Physicians' Desk Reference^® ^(PDR^®^) [13].

In order to collect randomized controlled trials (RCTs) on safety and efficacy of ophthalmic drugs in the paediatric population, a bibliographic search for ophthalmological therapy in children aged up to 18 years in the MEDLINE (1967 - December 2010), EMBASE (1975 - December 2010), and Cochrane Central Register of Controlled Trials (1967 - December 2010) databases was performed. The MeSH search terms and additional keywords used in the search strategy were: child/infant/newborn/adolescent, ophthalmology, drug therapy, and randomized controlled trials, limiting the results to human. To make the search more complete, the terms were searched for both in the database dictionaries and through the free text search option that covered the articles' titles and abstracts. All the references retrieved were collected and analyzed using the software program Reference Manager, version 11 (Institute for Scientific Information, Berkeley, California). The titles and abstracts were screened independently by two reviewers (FF and AC) to assess the relevance of the studies. Contrasting results were reviewed by a third person (MB).

We also searched for guidelines concerning paediatric ophthalmology management in MEDLINE and EMBASE, in the National Guidance Clearinghouse, National Library of Guidelines Specialist Library, National Institute for Clinical Excellence (NICE), Australian National Health and Medical Research Council, Canadian Medical Association InfoBase and New Zealand Guidelines Group databases, and on the American Academy of Pediatrics, Canadian Pediatric Society, and Royal College of Pediatrics websites.

In addition, a search for paediatric clinical trials on ocular medications in the World Health Organization's International Clinical Trials Registry Platform (ICTRP) [14], the ClinicalTrials.gov registry [15], and the International Standard Randomized Controlled Trial Number Register (ISRCTN) [16] was performed in order to find which of these drugs are under paediatric investigation. Furthermore, the list of paediatric investigation plans (PIPs) approved by EMA [17], the "List of the active substances included in the work-sharing procedure in accordance with Articles 45 and 46 of the European Paediatric Regulation [18], and the FDA's "Table of Medicines with new paediatric information", a list of drugs approved for paediatric use resulting from the paediatric clinical trials performed in response to paediatric legislative initiatives [19], and the updated priority list for studies into off-patent paediatric medicinal products [20], were also consulted in order to assess if there is a gap between research and clinical practice.

## Results

### Quantitative analysis

A total of 197 ocular medications were reported in the 2010 ATC index, respectively, 88 (45%), 63 (31%), and 41 (21%) of which were marketed in Italy, the UK, and the USA.

In all, 68 (35%) single drugs are licensed for paediatric use in at least one considered country, while 23 drugs (12%) were marketed in all three countries. More specifically, in Italy 43 single drugs (48% of those marketed) had a paediatric license, while 39 (64%) did in the UK and 22 (54%) did in the USA. Only 13 drugs were marketed with a paediatric license in all the countries (Table 1). Only 3 licensed drugs appear in the World Health Organization (WHO) list of paediatric essential drugs. Tetracycline as 1% eye ointment and adrenaline as 2% eye drops, considered essential drugs for children, were not licensed for paediatric use in any country.

**Table 1:** Paediatric licensing status and number of RCTs related to ocular medications

Pharmaco-therapeutic Group	Drug name	Licence Status			RCTs	
		
		IT	UK	USA	Published	Non-published
**ANTI-ALLERGY MEDICATIONS**		14/12*	8/8*	11/10*	**34**	**5**

**Anti-histamine agent**	Azelastine	≥ 4 yrs	≥ 12 yrs	≥ 3 yrs	4	-
	
	Emedastine	≥ 3 yrs	≥ 3 yrs	≥ 3 yrs	1	-
	
	Epinastine	≥ 12 yrs	≥ 12 yrs	≥ 3 yrs	-	-
	
	Ketotifen	≥ 3 yrs	≥ 3 yrs	≥ 3 yrs	5	3
	
	Levocabastine	all	NA**	NA	6	-
	
	Olopatadine	≥ 3 yrs	≥ 3 yrs	≥ 3 yrs	4	1

**Mast cell stabilizer**	Spaglumic acid	all	NA	NA	-	-
	
	Lodoxamide	all	≥ 4 yrs	> 2 yrs	1	-
	
	Nedocromil sodium	≥ 6 yrs	≥ 6 yrs	≥ 3 yrs	5	-
	
	Sodium cromogligate	ns**	all	NA	6	-
	
	Pemirolast	NA	NA	≥ 3 yrs	-	-

**Decongestant (Sympathomimetic agent)**	Naphazoline	≥ 10 yrs	NA	nl	-	1
	
	Oxymetazoline	≥ 3 yrs	NA	≥ 6 yrs	-	-
	
	Tetryzoline	≥ 3 yrs	NA	≥ 6 yrs	-	-

**ANTI-ALLERGY COMBINATIONS**		**3/3**	**1/1**	**2/2**	**0**	**0**

**Anti-histamine agent + Decongestant**	Antazoline + Xylometazoline	NA	≥ 5 yrs	NA	-	-
	
	Chlorpheniramine + Tetryzoline	≥ 3 yrs	NA	NA	-	-
	
	Pheniramine + Tetryzoline	≥ 3 yrs	NA	NA	-	-

**Mast cell stabilizer + Decongestant**	Cromoglicate + Tetryzoline	≥ 3 yrs	NA	NA	-	-

**Astringent + Decogestant**	Zinc sulfate + Tetryzoline	NA	NA	≥ 6 yrs	-	-

**Decogestant + Lubricants**	Tetryzoline + Povidone + Dextran 70 + Polyethylene glycol 400	NA	NA	≥ 6 yrs	-	-

**ANTI-GLAUCOMA MEDICATIONS**		**23/6**	**14/1**	**6/1**	**4**	**3**

**Beta-blocker**	Timolol	> 1 m	nl**	nl	4	2

**Carbonic anhydrase inhibitor**	Dorzolamide	all	nl	ns	-	

**Sympathomimetic agent (selective α**_**2 **_**- agonist)**	Apraclonidine	≥ 12 yrs	≥ 12 yrs	nl	-	-
	
	Brimonidine	≥ 12 yrs	NA	≥ 2 yrs	-	1
	
	Clonidine	ns	NA	NA	-	-

**Parasympathomimetic (colinergic) agent**	Aceclidine	≥ 3 yrs	NA	NA	-	-
	
	Pilocarpine	≥ 3 yrs	nl	Nl	-	-

**ANTI-GLAUCOMA COMBINATIONS**		**2/1**	**1/0**	**2/2**	**1**	**0**

**Beta-blocker + Carbonic anhydrase inhibitor**	Timolol + Dorzolamide	≥ 2 yrs	nl	≥ 2 yrs	1	-

**Beta-blocker + Sympathomimetic agent**	Timolol + Brimonidine	nl	NA	≥ 2 yrs	-	-

**ANTI-INFLAMMATORY MEDICATIONS**		**16/8**	**8/5**	**5/2**	**16**	**6**

**Non-Steroidal Anti-Inflammatory Drug (NSAID)**	Diclofenac	≥ 3 yrs	nl	nl	4	1
	
	Indomethacin	≥ 3 yrs	NA	NA	-	-
	
	Ketorolac	NA	nl	≥ 3 yrs	3	-

**Steroid agent**	Betamethasone	NA	all	NA	-	-
	
	Desonide	> 1 m	NA	NA	-	-
	
	Dexamethasone	> 1 m	all	nl	6	2
	
	Fluorometholone	≥ 2 yrs	≥ 2 yrs	≥ 2 yrs	3	1
	
	Hydrocortisone	> 1 m	all	NA	-	-
	
	**Prednisolone**	NA	all	nl	-	2
	
	Clobetasone	>1 m	NA	NA	-	-

**Steroid agent + Decongestant**	Fluorometholone + Tetryzoline	≥ 2 yrs	NA	NA	-	-
	
	Clobetasone + Tetryzoline	ns	NA	NA	-	-

**ANTI-INFECTIVE MEDICATIONS**		**21/13**	**9/9**	**10/7**	**27**	**3**

**Antibacterial agent**	Chloramphenicol	≥ 3 yrs	all	NA	7	-
	
	Fusidic acid	ns	all	NA	3	-
	
	Propamidine isetionate	NA	all	NA	-	-

**Aminoglycoside**	**Gentamycin**	≥ 3 yrs	all	>1yr	2	1
	
	Neomycin	NA	all	NA	-	-
	
	Netilmycin	> 1 m	NA	NA	-	-
	
	Tobramycin	≥ 1 yr	NA	nl	3	-

**Quinolone**	Ciprofloxacin	all	≥ 1 yr	≥ 1 yr	2	-
	
	Gatifloxacin	NA	NA	≥ 1 yr	-	
	
	Levofloxacin	≥ 1 yr	≥ 1 yr	≥ 1 yr	2	-
	
	Lomefloxacine	≥ 1 yr	NA	NA	-	-
	
	Moxifloxacin	≥ 1 m	nl	≥ 1 yr	3	
	
	Ofloxacin	nl ophtalmia neonatorum	> 1m	≥ 1 yr	1	-

**Antiviral agent**	**Acyclovir**	all	all	nl	-	-
	
	Idoxuridine	≥ 3 yrs	NA	NA	-	-
	
	Trifluridine	all	NA	≥ 6 yrs	-	-

**Other anti-infective agent**	Povidone - Iodine	> 1 m	NA	nl	4	2

**ANTIBACTERIAL COMBINATIONS**		**9/2**	**6/3**	**4/1**	**3**	**0**

**Antibacterials**	Polimyxin B + Trimethoprim	NA	all	> 2 ms	1	-
	
	Polimyxin B + Bacitracin	NA	all	nl	1	-
	
	Neomycin + Polymyxin B + Gramicidin	NA	≥ 2 yrs	nl	-	-
	
	Neomycin + Chloramphenicol	ns	NA	NA	-	-

**Antibacterial + Steroid**	Neomycin + Polymyxin B + Dexamethasone	nl	all	≥ 2 yrs	-	-
	
	Neomycin + Polymyxin B + Hydrocortisone	NA	NA	ns	-	-
	
	Neomycin + Chloramphenicol + Hydrocortisone	ns	NA	NA	-	-
	
	Neomycin + Prednisolone	ns	all	NA	-	-
	
	Neomycin + Fluocinolone	ns	NA	NA	-	-
	
	Neomycin + Betamethasone	NA	all	NA	1	-
	
	Tobramycin + Dexamethasone	nl	NA	≥ 2 yrs	-	-
	
	Prednisolone + Sulphacetamide	NA	NA	≥ 6 yrs	-	-
	
	Tobramycin + Fluorometholone	NA	NA	≥ 2 yrs	-	-

**Antibacterial + Steroid + Decongestant**	Neomycin + Gramicidin + Tetryzoline + Dexamethasone	≥ 3 yrs	NA	NA	-	-

	Betamethasone + Sulphacetamide + Tetryzoline	≥ 2 yrs	NA	NA	-	-

**MYDRIATIC/CYCLOPLEGIC MEDICATIONS**		**7/4**	**6/5**	**2/1**	**55**	**3**

**Antimuscarinic agent**	Cyclopentolate	≥ 3 yrs	> 3 ms	all	14	-
	
	Homatropine	ns	> 3 ms	nl	-	-
	
	Tropicamide	> 1 m	all	NA	10	-
	
	**Atropine**	ns	> 3 ms (nl uveitis)	NA	31	3
	
	Ibopamine	all	NA	NA	-	-

**Decongestant (Sympathomimetic agent)**	Phenylephrine	≥ 12 yrs	All (nl 10% drops)	NA	2	-

**PERI-OPERATIVE MEDICATIONS**		**2/0**	**4/4**	**1/0**	**4**	**0**

**Local anaesthetic**	Lidocaine	nl	all	nl	2	-
	
	Oxybuprocaine	ns	all	NA	1	-
	
	Proxymetacaine	NA	all	NA	-	-
	
	**Tetracaine**	NA	all	NA	1	-

**LUBRICANTS AND ASTRIGENTS**		**5/0**	**10/5**	**4/0**	**0**	**0**

**Ocular lubricant and astringent**	Polyvinyl alcohol	Ns	all	NA	-	-
	
	Carmellose sodium	ns	all	NA	-	-
	
	Hydroxyethylcellulose	NA	all	NA	-	-
	
	Paraffin	NA	all	NA	-	-
	
	Sodium hyaluronate	ns	all	NA	-	-
	
	Hypromellose	NA	all	nl	-	-

**LUBRICANT COMBINATIONS**		**0/0**	**2/2**	**6/2**	**0**	**0**

**Lubricants**	Hypromellose + Glycerin	NA	NA	all	-	-
	
	Hypromellose + Dextran 70	NA	all	nl	-	-
	
	Hypromellose + Glycerin + Polyethylene glycol 400	NA	NA	≥ 6 yrs	-	-

**Lubricant + Steroid agent**	Hypromellose + Dexamethasone	NA	all	NA	-	-

**OTHER OCULAR MEDICATIONS**		**2/1**	**2/2**	**2/1**	**0**	**0**

**Hypertonic agent**	Sodium chloride	NA	all	Nl	-	-

**Ocular diagnostic agent**	Fluorescein	ns	all	NA	-	-

**Topical immunomodulator**	Cyclosporine 0.05%	NA	NA	≥ 16 yrs	-	-

**Other ocular agent**	Heparin	> 1 m	NA	NA	-	-

**TOTAL SINGLE DRUGS**	**68**	**88/43**	**61/39**	**41/22**	**140**	**20**

**TOTAL COMBINATIONS**	**29**	**16/7**	**10/6**	**14/7**	**4**	-

NOTE: Only drugs with a paediatric licence at least in one country are listed. The drugs in bold are listed in the WHO model list of essential medicines for children.

* N° drugs marketed/N° drugs marketed with paediatric licence

** ns: not specified; nl: not licensed for paediatric use; NA: not authorised

Fifteen single drugs and six combinations (mainly anti-infective, anti-inflammatory, and anti-allergy medications) were licensed for paediatric use only in Italy, while 16 single drugs and 8 combinations were licensed only in the UK (mainly local anaesthetics and lubricants), and 2 single drugs and 8 combinations only in the USA (mainly anti-infective medications). Almost all anti-allergy medications and combinations had a paediatric license in all three countries, while no local anaesthetics are licensed for paediatric use in Italy and USA and no NSAIDs are in the UK.

Wide differences were found in the age groups for which the drugs were licensed and only for 6 drugs the age range is the same or similar in all countries.

### Qualitative analysis

#### Bibliographic search

The bibliographic search produced 158 RCTs on 69 single drugs and combinations, involving a total of 18,816 children (Table 2). The percentage of drugs licensed for paediatric use with at least one RCT ranged between 51% in Italy and 55% in the USA. No published RCTs were found for 11 (48%) ocular medications licensed for paediatric use in all three countries.

**Table 2:** Summary of retrieved RCTs on the use of ocular medications in the paediatric population

Pharmaco-therapeutic Group	Drug name	Formulation	N° RCTs	N° Children	Age range
**MYDRIATIC/CYCLOPLEGIC MEDICATIONS**			**74 (35%)**		

**Antimuscarinic agent**	Atropine	eye drops 1%	31	3530	all
	
	Cyclopentolate	eye drops 0.5%	2	28	≤ 13 yrs
		
		eye drops 1%	11	181	≤ 16 yrs
	
	Tropicamide	eye drops 1%	9	348	all
	
	Pirenzepine	ophthalmic gel 1%	3	276	6 - 12 yrs
	
	Cyclopentolate/Tropicamide	eye drops 1%/1%	6	176	all

**Sympathomimetic agent**	Phenylephrine	eye drops 2.5%	1	10	≤ 1 m

**Antimuscarinic agent + Sympathomimetic agent**	Tropicamide/Phenylephrine	eye drops 1%/2.5%	3	92	≤ 1 m
		
		eye drops 0.5%/2.5%	2	51	≤ 8 yrs
		
		eye drops 0.5%/0.5%	1	12	3-11 yrs
	
	Cyclopentolate/Phenylephrine	eye drops 1%/2.5%	2	30	≤ 6 yrs
		
		eye drops 0.2%/1%	3	99	≤ 1 m

**ANTI-INFECTIVE MEDICATIO NS**			**51 (24%)**		

**Antibacterial agent**	Chloramphenicol	eye drops 0.5%	7	1664	≤ 12 yrs
	
	Azithromycin	eye drops 1%	1	335	≥ 1 yr
	
		eye drops 1.5%	2	542	≥ 1 yr
	
	Tetracycline	eye drops 1%	2	518	1- 10 yrs
	
		eye ointment 1%	2	218	≥ 6 ms
	
	Besifloxacin	eye suspension 0.6%	3	1124	≥ 1yr
	
	Fusidic acid	eye drops 1%	3	594	≤ 2 yrs
	
	Moxifloxacin	eye drops 0.5%	3	645	all
	
	Tobramycin	eye drops	3	741	≤ 12 yrs
	
	Ciprofloxacin	eye drops 0.3%	2	193	≤ 12 yrs
	
	Levofloxacin	eye drops 0.5%	2	106	1-16 yrs
	
	Gentamycin	eye ointment	2	117	≤ 12 yrs
	
	Erythromycin	eye drops	1	110	≤ 1 m
	
		eye ointment	1	24	≤ 1 yr
	
	Ofloxacin	eye drops 0.3%	1	23	≥2 yrs
	
	Oxytetracycline	eye drops 1%	1	450	≤ 1 m
	
	Sulphacetamide	eye drops 10%	1	14	≤ 1 m

**Antibacterials combinations**	Polymixin B/Oxytetracycline	eye ointment	2	132	2-10 yrs
	
	Polymixin B/Bacitracin	eye ointment	1	66	≥ 1 m
	
	Polymixin B/Trimethoprim	eye drops	1	28	all

**Antibacterial agent + NSAID**	Gentamycin/Diclofenac	eye drops	1	12	≤ 12 yrs

**Antibacterial agent + Steroid agent**	Neomycin/Betamethasone	eye drops	1	12	≤ 12 yrs
	
	Tobramycin/Dexamethasone	eye drops	1	28	4-10 yrs

**Antifungal agents**	Miconazole	eye suspension 1%	1	12	≥ 15 yr
	
	Econazole/Miconazole	eye suspension 1%/1%	1	7	≥ 15 yr

**Other anti-infective eye preparation**	Povidone-iodine	eye drops 2.5%	4	3132	≤ 1 yr
	
	Silver nitrate	eye drops 1%	1	450	≤ 1 m

**ANTI-ALLERGY MEDICATIONS**			**31 (15%)**		

**Anti-histamine agent**	Levocabastine	eye suspension 0.5%	6	174	≥ 3 yrs
	
	Ketotifen	eye drops 0.025%	5	522	≥ 3 yrs
	
	Olopatadine	eye drops 0.2%	4	99	≥ 3 yrs
	
	Azelastine	eye drops 0.02%	4	132	≥ 4 yrs
	
	Bepotastine	eye drops 1%	1	36	≥ 10 yrs
	
		eye drops 1.5%	1	36	≥ 10 yrs
	
	Emedastine	eye drops 0.05%	1	-	3-16 yrs

**Mast cell stabilizer**	Lodoxamide	eye drops 0.1%	1	15	≥ 6 yrs
	
	Cromoglycate	eye drops 2%	4	128	≥ 4 yrs
		
		eye drops 4%	1	30	≥ 16 yrs
	
	Nedocromil	eye drops 2%	3	85	≥ 4 yrs

**ANTI-INFLAMMATORY MEDICATIONS**			**18 (9%)**		

**Corticosteroid**	Dexamethasone	eye drops 0.1%	6	159	all
	
	Fluorometholone	eye drops 0.1%	3	52	≤ 10 yrs
	
	Rimexolone	eye drops 1%	1	22	4 - 8 yrs

**NSAID**	Diclofenac	eye drops 0.1%	4	93	≥ 2 yrs
	
	Ketorolac	eye drops 0.5%	3	70	≤ 12 yrs
	
	Flurbiprofen	eye drops	1	50	≥ 5 yrs

**ANTIGLAUCOMA MEDICATIONS**			**10 (5%)**		

**Beta-blocker**	Timolol	eye drops 0.25%	2	44	7-13 yrs
		
		eye drops 0.5%	1	12	≥ 14 yrs
		
		gel-forming solution 0.25%	1	35	≤ 6yrs
		
		gel-forming solution 0.5%	1	36	≤ 6yrs
	
	Betaxolol	eye suspension 0.25%	1	52	≤ 6 yrs
	
	Levobetaxolol	eye suspension 0.5%	1	46	≤ 6 yrs

**Carbonic anhydrase inhibitor**	Brinzolamide	eye suspension 1%	1	32	≤ 6 yrs
	
	Dorzolamide	eye drops 2%	1	56	≤ 6 yrs

**Acetylcholinesterase inhibitor**	Echothophate iodide	eye drops	1	20	-

**PERI-OPERATIVE MEDICATIONS**			**18 (9%)**		

**Local anaesthetic agent**	Bupivacaine	subconjuntival infiltration	2	38	5-10 yrs
		
		eye drops 0.5%	1	17	3-6 yrs
	
	Proparacaine	eye drops 0.5%	3	58	≤ 1 m
	
	Lidocaine	eye drops 2%	1	10	3 - 14 yrs
		
		ophthalmic gel 2%	1	24	3-12 yrs
	
	Amethocaine	eye drops 0.5%	2	45	2 - 8 yrs
	
	Levobupivacaine	eye drops	1	13	1 - 16 yrs
	
	Oxybuprocaine	eye drops 0.4%	1	20	3-8 yrs
	
	Tetracaine	eye drops 1%	1	44	1-12 yrs
	
	Sucrose	eye drops	1	11	≤1 m

**Chemotherapeutic agents**	Mitomicyn C	eye drops 0.02%	1	10	≥ 6 yrs
		
		ocular injection 0.4%	1	7	≥ 6 yrs
	
	5-fluorouracil	ocular injection	1	4	≤ 12 yrs
	
	Mitomicyn C/5-fluorouracil	ocular injection	1	4	≤ 12 yrs

**OTHER DRUGS**			**7 (3%)**		

**Vernal keratoconjunctivitis**	Cyclosporine	eye drops 2%	1	14	5-16 yrs
		
		eye drops 1.25%	1	20	5-14 yrs
		
		eye drops 1%	1	32	5-14 yrs
	
	Mipragoside	ophthalmic gel 0.5%	1	12	5-20 yrs

**ROP therapy**	Bevacizumab	intravitreal injection	1	7	≤ 1 m

**Esotropia**	Botulinum toxin	ocular injection	1	27	6-12 yrs

**Dacryocystitis**	Herba houttuyaniae	eye drops	1	268	≤ 1 m

**TOTAL (69 single drugs & combinations)**			**209**	**18,816**	

NOTE: the total is higher than the sum of the RCTs (158) because some drugs were tested in more than one trial. The references to RCTs are available upon request to the corresponding author.

In all, 74 retrieved RCTs (35%) regarded mydriatic/cycloplegic medications, mainly antimuscarinic agents. In particular, 31 RCTs involving 3,530 children belonging to all age groups studied atropine as eye drops 1%, a drug licensed for paediatric use only in the UK (≥ 3 months). In addition, 3 studies were available on pirenzepine, a drug not licensed for paediatric use in any country.

Regarding the treatment of allergic conjunctivitis, 49 (23%) RCTs on 11 drugs were found: 22 studies involved 6 anti-histamine agents (azelastine, bepotastine, emedastine, ketotifen, levocabastine, and olopatadine) tested in children ≥ 3 years, and 9 RCTs involved 3 mast cell stabilizers, such as lodoxamide, cromoglicate, and nedocromil, in children ≥ 4 years. Bepotastine is the only drug unlicensed for paediatric use in all considered countries.

A total of 43 RCTs (21%) concerned 20 antibacterial agents and their combinations, 8 of them (40%) not licensed for paediatric use in any country considered, such as the fluoroquinolone besifloxacin as eye suspension 2%, tested in 3 RCTs in children older than one year. Among the six combinations studied 3 were licensed for paediatric use in the UK and 2 in the USA, while none in Italy. In addition, the anti-infective agent povidone-iodine, licensed for use in children older than 1 month, was studied only in Italy in 3 RCTs.

Among the medications commonly used in ophthalmic surgical procedures (e.g. strabismus surgery) there were 7 local anaesthetics (proparacaine, not licensed for paediatric use in any country was the drug most studied), 3 steroids (dexamethasone, fluorometholone, and rimexolone), and 3 NSAIDs (diclofenac, ketorolac, and flurbiprofen).

Ten RCTs regarded anti-glaucoma agents: 7 were on 3 beta-blockers, 2 on carbonic anhydrase inhibitors, and the last one on an acethylcholinesterase inhibitor, echothophate iodide. The most studied drug is timolol, a beta-bloker licensed for use in children older than 1 month only in Italy, as well as the carbonic anhydrase inhibitor dorzolamide.

Phenylephrine, the only decongestant agent studied, licensed for paediatric use in all countries considered, was involved in 11 RCTs, in which it was used in combination with a mydriatic/cyclopegic agent for eye examinations in children. In one RCT involving 10 neonates, phenylephrine was used alone.

#### Guidelines

Eight guidelines on pharmacological management of eye diseases in children were found: they addressed acute bacterial conjunctivitis [21], amblyopia [22,23], strabismus [24], glaucoma [25], retinopathy of prematurity (ROP) [26,27], and prophylaxis of neonatal ophthalmia [28] (Table 3). Those concerning screening methods for diagnosing eye diseases in the paediatric population without drug use were not reported.

**Table 3:** Summary of guidelines on pharmacological therapy of ocular disease in the paediatric population

**Ref**.	Organisation	Title	Disease	Quality of evidence	Treatment (*Licensing status*)	Country	Year
[41]	National Guideline Clearinghause (NGC)	Guidelines for the treatment and management of acute bacterial conjunctivitis in children and adults.	Acute bacterial conjunctivitis	I	Topical antibiotic therapy: • Norfloxacin 0.3% (*nl*) • Ciprofloxacin 0.3% • Ofloxacin 0.3% • Levofloxacin 0.5% (*nl UK, nl USA*) • Lomefloxacin 0.3% • Moxifloxacin 0.5% (*nl UK*) • Gatifloxacin 0.3% (*nl IT, nl UK*) • Chloramphenicol 0.5% *(nl USA)* • Sulfacetamide Sodium 10% *(nl)* • Erythromycin 0.5% *(nl)* • Gentamicin Sulfate 0.3% *(nl)* • Trimethoprim Sulfate/Polymyxin B 10000 U/1 *mg/mL (nl IT)* • Fusidic acid 0.1% *(nl IT, nl USA)* • Tobramycin 0.3% *(nl UK, nl USA)* • Povidone-iodine 1.25% *(nl UK, nl USA)* • Bacitracin *(nl)* Ocular steroids and steroid-antibiotic: • Prednisolone *(nl IT, nl USA)* • Fluorometholone 0.1%/sulfacetamide sodium 10% *(nl)* • Fluorometholone 0.1% • Neomycin/polymyxin B/dexamethasone 0.1% *(nl IT)* • Gentamicin 0.3%/prednisolone acetate 0.1% *(nl)* • Tobramycin 0.3%/dexamethasone 0.1% *(nl)*	USA	2005
[34]	Canadian Paediatric Society	Recommendations for the prevention of neonatal ophthalmia	Prophylaxis to prevent neonatal ophthalmia due to N gonorrhoeae	III	• Silver nitrate 1% eye drops *(nl)* • Erythromycin 0.5% ointment *(nl)* • Tetracycline 1% ointment *(nl)*	Canada	2002 (Rev. 2009)
[29]	Moore W. and Nischal K.K.	Pharmacologic management of glaucoma in childhood	Glaucoma	I	• Β-Blockers: Betaxolol 0.25% *(nl)* • Carbonic Anhydrase Inhibitors: Dorzolamide 2% *(nl UK, nl USA)* • Prostaglandin Analogs: Latanoprost *(nl)*, Travoprost *(nl)*, Bimatoprost *(nl)* • Adrenoceptor Agonists: Brimonidine *(nl UK)*, Apraclonidine *(nl)* • Parasympathomimetics: Pilocarpine *(nl UK, nl USA)*	UK	2007
[39]	Royal College of Ophthalmologists	Guidelines for the management of amblyopia	Ambliopia	III	• Refractive correction (glasses) • Patching: from 2 to 6 hours per day • Atropine *(nl IT, nl USA)*	UK	2006
[6]	National Guideline Clearinghause (NGC)	Best evidence statement (BESt). Treatment of amblyopia in children.	Amblyopia	I	• Refractive correction (glasses) • Atropine: 1 drop/day, 2 - 7 days per week *(nl IT, nl USA)* • Patching: from 2 to 6 hours per day	USA	2007
[38]	Royal College of Ophthalmologists	Guidelines for the management of strabismus in childhood	Strabismus	III	• Surgical interventions • Refractive correction (glasses) • Miotics (not specified)	UK	2007
[46]	The Brazilian Society of Pediatrics, Brazilian Council of Ophthalmology, Brazilian Society of Pediatric Ophthalmology	Brazilian guidelines proposal for screening and treatment of retinopathy of prematurity (ROP)	Retinopathy of prematurity (ROP)	III	Surgical interventions+post surgical treatment with topical steroids/antibiotics (not specified)	Brazil	2007
[40]	Royal College of Ophthalmologists, Royal College of Paediatrics and Child Health, British Association of Perinatal Medicine & BLISS	Guideline for the Screening and Treatment of Retinopathy of Prematurity	Retinopathy of prematurity (ROP)	III	Screening examination with Cyclopentolate 0.5%/Phenylephrine 2.5% combination: 1drop each in 2 to 3 doses, each 5 minutes apart, 1 hour prior to examination (*nl*)	UK	2008

NOTE: nl: not licensed for paediatric use; IT: Italy; UK: United Kingdom; USA: United States of America

Five guidelines (2 regarding ROP, 2 regarding amblyopia, and 1 regarding strabismus) recommended drug use only for screening or post-surgical therapy, and not for the pharmacological management of the disease in childhood.

Almost all of the drugs listed in the guidelines are not licensed for use in children in any country considered, especially for prophylaxis of neonatal ophthalmia (no drug licensed), for the medical management of childhood glaucoma (5 out of 8 drugs are unlicensed) and acute bacterial conjunctivitis (8 out of 22 drugs are unlicensed). The authors indicated that all these drugs are generally used in a off label manner and that the majority of data on these medications are from adult studies.

Finally, no guidelines on the pharmacological treatment of allergic conjunctivitis were found.

#### Search for the paediatric RCTs in registries

A search performed in the World Health Organization's International Clinical Trials Registry Platform (ICTRP), the ClinicalTrials.gov registry, and the International Standard Randomized Controlled Trial Number Register (ISRCTN) found 46 ocular medications currently under paediatric investigation in 62 RCTs (56% of which completed). Cyclosporin, an immunosuppressant agent, and bevacizumab, a humanized monoclonal antibody, were the drugs involved in the most studies: 7 RCTs testing cyclosporine in the treatment of keratoconjunctivitis (4), dry eye syndrome (2), and pterygia (1), and 4 RCTs on bevacizumab in the treatment of neovascular glaucoma in children > 3 years (all 3 completed) and in ROP in neonates > 5 months (1 ongoing RCT).

Among the drugs that had the most ongoing studies were also two anti-hystamine drugs, ketotifen and bepotastine, and the antibacterial moxifloxacin: these were tested in 3 RCTs each for the treatment of allergic or bacterial conjunctivitis in children.

#### EMA/FDA viewpoint

Although no ophthalmologic drugs are found in the EMA's priority list for studies into off-patent paediatric medicinal products at this time, the EMA Paediatric Committee (PDCO) adopted opinions on PIPs for 12 ocular medications, with the aim to generate the necessary quality, safety, and efficacy data to support the authorization of these medicines for use in children.

Four drugs, cysteamine, latanoprost, voclosporin and the recombinant human monoclonal antibody to human interleukin 17A received a go-ahead for a PIP, while one, travoprost/brinzolamide, was refused it. In four cases, one involving the anti-inflammatory agent bromfenac, one a new drug, ocriplasmin, for the treatment of symptomatic focal vitreomacular adhesion, and two the vascular endothelial growth factor inhibitors, ranimizumab and pegaptanib, a waiver was granted in all age groups on the grounds that the specific medicinal product does not represent a significant therapeutic benefit or because the disease or condition for which the product is intended does not occur in the specified paediatric subset(s). Finally, 2 steroid drugs, dexamethasone and triamcinolone, were refused the granting of a product-specific waiver on the grounds that the clinical studies cannot fulfil a therapeutic need of the paediatric population.

By consulting the "List of the active substances included in the work-sharing procedure in accordance with Articles 45 and 46 of the European Paediatric Regulation, no additional data or information on their use in the paediatric population resulted to be submitted or requested to authorise the paediatric use of any ocular medicinal product.

Twenty-six ocular medications were found in the Food and Drug Administration (FDA)'s "Table of Medicines with New Paediatric Information", a list of drugs approved for use in the paediatric population resulting from the paediatric clinical trials performed in response to paediatric legislative initiatives. Ten (38%) were anti-allergy medications, 8 (31%) were anti-glaucoma medications (6 of which were not yet licensed for paediatric use in the USA), and 5 were antibacterials and combinations. The last three agents were triamcinolone (steroid agent), lidocaine (local anaesthetic agent), and a hypromellose combination (lubricant). These drugs included approved information on use in the paediatric population resulting from the paediatric clinical trials performed in response to paediatric legislative initiatives.

## Discussion

This article reviews ocular medication use in children, providing a summary of their licensing status in Italy, the UK, and the USA and analyse the amount of available studies testing these medicines in the paediatric population. Most of the drugs listed have only recently obtained paediatric use approval and are now widely prescribed for children by a growing number of clinicians [29]. However, for most of these drugs wide differences in the licensed age groups were found and only a few are available in all three countries. Even if the Paediatric Regulation in EU and USA specifically aims at giving children the same access to authorised medicinal products suitable for their use, the age approval and occasionally the approach towards certain therapeutic problems is under the direct responsibility of National Authorities, so differences in drug licensing procedure between countries remain. There is therefore a need for evidence-based harmonization of drug licenses in order to guarantee equal drug availability and access [30].

Furthermore, many ocular medications commonly used in children still do not have paediatric dosing and safety labelling information in any country. For example, almost for all glaucoma medications (such as prostaglandin analogues and carbonic anhydrase inhibitors), paediatric use is labelled "not recommended".

At this time no paediatric RCTs were available for several ocular medications. When available, the studies were often limited to small case series and case reports, so more extensive controlled trials will be needed to confirm their safety and efficacy also in paediatric population. On the contrary, evidence on efficacy was found for drugs that were not licensed for children, such as tetracycline and bupivacaine.

In spite of the fact that no ophthalmologic drugs are found in the EMA's priority list, several drugs were recently studied in paediatric clinical trials in the European countries and the USA. In particular, the ongoing research is examining the potential use of intravitreally injected anti-VEGF drugs, such as bevacizumab, successfully used in adults with diabetic retinopathy or age-related macular degeneration (AMD), a cause of a severe vision loss among the aging population in many western countries [31,32]. These drugs could now also be used in paediatric vitreoretinal diseases, as shown by recent studies on intravitreal injection of bevacizumab for the treatment of ROP, the leading cause of childhood blindness [33-37].

Moreover, the available guidelines on the pharmacological management of eye diseases in the paediatric population often recommend the use of medications not licensed or investigated in children, especially for the management of glaucoma (such as prostagliandin analogs) or acute bacterial conjunctivitis (such as steroids and antibiotics combinations). An effort to stimulate research and clinical development is therefore needed also for them, in order to guarantee medicines that have been proven to be of benefit also in paediatric patients.

Many good ethical and economical reasons exist for limiting paediatric clinical trials, while guaranteeing appropriate conclusions. Because of the characteristics of the paediatric population, limited information is also available regarding the side effects related to ocular medication use in children [38]. As the number and variety of ocular medications has increased and the number of clinicians involved in their prescription has grown, the risk of systemic adverse reactions may also increase [39,40]. When prescribing ocular medications in children, physicians should therefore carefully consider their risk/benefit profile, referring to details of labelling for paediatric use, such as the age of the child for whom the drug is approved, and be aware of their potentially serious systemic side effects [5].

Some strategies for reducing systemic absorption and toxicity should be followed whenever possible. First of all, the lowest available dosage of medication necessary to achieve a therapeutic benefit while minimizing risk should be used. Secondly, since different formulations may have different degrees of systemic absorption, formulations with lower systemic absorption, which may be more suitable for use in children, should be used. Ophthalmic gel or ointment, for example, has been found to have reduced systemic absorption compared to the ophthalmic solution [28]. In addition, paediatric patients should be monitored closely during and after treatment for local and systemic side effects [29].

The present findings suggest that access to, and rational use of, ocular medications in the paediatric population continue to present a considerable challenge. Paediatric clinical trials are important for defining how infants and children respond to medications and for identifying age-specific toxic effects [41]. While recent legal and economic incentives in both Europe and the USA stimulate research to obtain more data regarding dosing, efficacy, and safety of medicines used in children, problems remain in obtaining adequate evidence [42]. In this context, there is a pressing need for further clinical research to improve the quality, efficacy, and safety of ocular medications offered to paediatric patients. Clinical research must be carried out using appropriate methodologies (e.g. study design, sample size, randomization, and blinding) [38] also (and in particular) in the paediatric ophthalmic area, where effective up-to-date treatments, and additional research and education on use in children, remain priorities [43].

## Conclusion

European and American legislation has established that children should have the same rights as adults to receive medicines that have been proven to be of benefit and that are unlikely to cause serious toxicity [44]. Even if the legislative initiatives in both Europe and the USA emphasizes the importance of large clinical trial in children, prioritizing the medicines to be studied on the basis of children's needs [45], differences between countries in drug licensing procedures, and occasionally in the approach towards certain therapeutic problems, may be quite significant [30]. A formulary containing common "paediatric" evidence-based safety and efficacy information could be a useful tool for improving the rational use of drugs in children and adolescents, harmonizing inter-country drug regulations and availability [46].

In addition, recommendations from high quality RCTs and systematic reviews, and effective knowledge translation strategies are essential to clinicians and policy makers in planning changes in practice that could ultimately improve patient- and system-related outcomes. All such considerations are priorities for an area, such as ophthalmic drug therapy in children, that is lacking evidence.

## Competing interests

The authors declare that they have no competing interests.

## Authors' contributions

FF carried out the bibliograhical search, screened studies for inclusion, performed data extraction and analysis, and drafted the manuscript; AC provided methodological advice; MB participated in the design of the study and revised the manuscript. All authors read and approved the final manuscript.

## Pre-publication history

The pre-publication history for this paper can be accessed here:

http://www.biomedcentral.com/1471-2431/12/8/prepub
